# EspF is crucial for *Citrobacter rodentium*-induced tight junction disruption and lethality in immunocompromised animals

**DOI:** 10.1371/journal.ppat.1007898

**Published:** 2019-06-28

**Authors:** Xue Xia, Yue Liu, Andrea Hodgson, Dongqing Xu, Wenxuan Guo, Hongbing Yu, Weifeng She, Chenxing Zhou, Lei Lan, Kai Fu, Bruce A. Vallance, Fengyi Wan

**Affiliations:** 1 Department of Biochemistry and Molecular Biology, Bloomberg School of Public Health, Johns Hopkins University, Baltimore, MD, United States of America; 2 Division of Gastroenterology, Department of Pediatrics, BC's Children's Hospital and Child and Family Research Institute, Vancouver, British Columbia, Canada; 3 Eudowood Division of Pediatric Infectious Diseases, Johns Hopkins University School of Medicine, Baltimore, MD, United States of America; 4 Jiangsu Province Key Laboratory for Molecular and Medical Biotechnology, College of Life Sciences, Nanjing Normal University, Nanjing, Jiangsu, PR China; 5 Institute of Molecular Precision Medicine, Xiangya Hospital, Central South University, Changsha, Hunan, PR China; 6 Department of Molecular Microbiology and Immunology, Bloomberg School of Public Health, Johns Hopkins University, Baltimore, MD, United States of America; 7 Department of Oncology, Sidney Kimmel Comprehensive Cancer Center, Johns Hopkins University, Baltimore, MD, United States of America; Tufts Univ School of Medicine, UNITED STATES

## Abstract

Attaching/Effacing (A/E) bacteria include human pathogens enteropathogenic *Escherichia coli* (EPEC), enterohemorrhagic *E*. *coli* (EHEC), and their murine equivalent *Citrobacter rodentium* (CR), of which EPEC and EHEC are important causative agents of foodborne diseases worldwide. While A/E pathogen infections cause mild symptoms in the immunocompetent hosts, an increasing number of studies show that they produce more severe morbidity and mortality in immunocompromised and/or immunodeficient hosts. However, the pathogenic mechanisms and crucial host-pathogen interactions during A/E pathogen infections under immunocompromised conditions remain elusive. We performed a functional screening by infecting interleukin-22 (IL-22) knockout (*Il22*^*-/-*^) mice with a library of randomly mutated CR strains. Our screen reveals that interruption of the *espF* gene, which encodes the Type III Secretion System effector EspF (*E*. *coli* secreted protein F) conserved among A/E pathogens, completely abolishes the high mortality rates in CR-infected *Il22*^*-/-*^ mice. Chromosomal deletion of *espF* in CR recapitulates the avirulent phenotype without impacting colonization and proliferation of CR, and EspF complement in *ΔespF* strain fully restores the virulence in mice. Moreover, the expression levels of the *espF* gene are elevated during CR infection and CR induces disruption of the tight junction (TJ) strands in colonic epithelium in an EspF-dependent manner. Distinct from EspF, chromosomal deletion of other known TJ-damaging effector genes *espG* and *map* failed to impede CR virulence in *Il22*^*-/-*^ mice. Hence our findings unveil a critical pathophysiological function for EspF during CR infection in the immunocompromised host and provide new insights into the complex pathogenic mechanisms of A/E pathogens.

## Author summary

Emerging evidence demonstrates that attaching/effacing (A/E) pathogens, the important causative agents of foodborne illnesses, cause more severe morbidity and mortality in immunocompromised hosts. However, the molecular and cellular mechanisms during A/E pathogen infections under immunocompromised conditions have remained obscure. More specifically, the critical host-pathogen interactions that account for the severe symptoms are not fully understood. Utilizing an immunodeficient mouse model for a functional screening, we have identified *E*. *coli* secreted protein F (EspF) as a critical virulence protein that plays a pivotal role in A/E pathogen *Citrobacter rodentium* (CR) infection-induced severe morbidity and mortality in immunocompromised animals. We have also revealed that the EspF-dependent disruption of tight junctions is crucial for the loss of colonic epithelial barrier function and the systemic spread of pathogens during infection. Our findings highlight the previously underappreciated pathophysiological relevance of EspF during A/E pathogen infections and increase our understanding of the sophisticated pathogenic mechanisms of foodborne diseases.

## Introduction

The human pathogens enterohemorrhagic *Escherichia coli* (EHEC) and enteropathogenic *E*. *coli* (EPEC) are the leading causative agents for foodborne diseases, which lead to severe economic burden, morbidity, and mortality worldwide [1,2,3,4]. EHEC, EPEC and their murine equivalent *Citrobacter rodentium* (CR) cause attaching and effacing (A/E) lesions, characterized by the attachment of the bacteria to host epithelial cells and the localized destruction of brush-border microvilli [5,6]. Notably, CR shares 66.7% of its genes and most pathogenic mechanisms with EPEC and EHEC [5,7,8,9,10,11]. Since human pathogens EPEC and EHEC do not infect mice well [3], CR infection in mice has been widely used as an animal model to study the pathogenic mechanisms of A/E pathogens. There are approximately 5,000 genes in each genome of EHEC, EPEC, and CR. However, only a miniscule portion of the encoded genes, including the best-characterized gene cluster *locus of enterocyte effacement* (LEE) pathogenicity island, has been functionally related to A/E pathogenicity [12,13]. It is therefore believed that a substantial amount of essential host-pathogen interactions during A/E pathogen infections *in vivo* remain poorly understood.

After the peak of CR colonization, the infection starts to clear and epithelial cells colonized by CR are shed into the intestinal lumen, with complete bacterial clearance occurring after 2–4 weeks of infection [2]. Wild-type C57Bl/6 mice and many immunocompetent strains show little or no mortality with CR infection; in striking contrast, interleukin-22 knockout (*Il22*^*-/-*^) mice display dramatically elevated morbidity and mortality rates during CR infection [14]. Patients with autoimmune polyendocrine syndrome type (carrying autoantibodies against IL-22) are more susceptible to various infections including A/E pathogens than healthy controls with normal IL-22 levels [15,16,17,18,19]. While it is believed that A/E pathogens adapt to the gastrointestinal tract and undergo a virulence change after adapting to the host [2], the virulence regulation of A/E pathogens, especially in immunocompromised or immunodeficient settings, remain elusive.

Besides serving as a selectively permeable barrier permitting the absorption of nutrients, the intestinal epithelium is critical for maintaining an effective defense against intraluminal toxins, antigens, and enteric flora [20]. In particular, tight junctions (TJs) in the apical domain of epithelial cells, comprised of transmembrane proteins (*e*.*g*. claudins and occludin) and cytosolic scaffold proteins (*e*.*g*. zonulae occludens [ZO] and cingulin), are crucial in preventing the entry of potentially harmful entities including bacterial pathogens [20]. A/E pathogens use the Type III secretion system (T3SS) to inject an array of virulence proteins (effectors) from the bacterial cytoplasm into the host cell cytoplasm, where they interfere with host cell signaling [3,6,21]. A/E pathogens are known to manipulate the signaling pathways related to TJ dynamics, opening this structure and triggering watery diarrhea in immunocompetent hosts [20,22]. For instance, the T3SS effectors EspF (*E*. *coli* secreted protein F), EspG (*E*. *coli* secreted protein G), Map (Mitochondrial associated protein), and NleA (Non-LEE encoded effector A) were shown to be required for A/E pathogen-induced disruption of the TJ strands and decreased transepithelial resistance (TER) in cultured monolayer cells [23,24,25,26,27,28]. TJ disruption by T3SS effectors injected by A/E pathogens has been studied extensively, mainly through a variety of *in vitro* approaches [23,24,25,26,27,29,30,31], with a few studies examining the relevance of EspF, Map, and NleA during infections in immunocompetent animals [22,28,32,33,34,35,36,37]. However, it remains poorly understood how these effectors are regulated when A/E pathogens adapt to the hosts, how they disrupt the TJ strands, and dampen intestinal epithelial barrier function during infections in the hosts.

Here through an *in vivo* functional screening by infecting *Il22*^*-/-*^ mice with wild-type CR or genetically manipulated mutant strains, we reveal that EspF, a multifunctional effector, plays a critical function in CR-caused severe morbidity and mortality in *Il22*^*-/-*^ mice by damaging TJ strands and disrupting colon epithelial integrity. Chromosomal deletion of *espF*, but not the other TJ-damaging effector genes *espG* and *map*, eliminated CR-induced severe lethality in *Il22*^*-/-*^ mice without affecting the colonization, proliferation, and clearance of CR in the host. Moreover, EspF deletion markedly attenuated CR infection-caused TJ disruption in the colon and systemic dissemination of CR in infected *Il22*^*-/-*^ animals. Our results therefore reveal an important pathophysiological function of EspF in host-pathogen interactions during infections and provide new insights into A/E pathogenic mechanisms.

## Results

### Generation of a CR mutant library by Tn5 transposon random mutagenesis

For a systemic analysis of the genes encoded in the CR genome, we utilized the Tn5 transposon-mediated random mutagenesis [38] to genetically manipulate the CR genome in wild-type CR (DBS 100 strain). The mutant CR strains, in which the Tn5 transposon was randomly inserted into the CR genome, were screened using kanamycin resistance (**S1 Fig**). After removing the mutants with growth defects, we generated a CR random mutant library with approximately 1,800 Tn5-interrupted CR mutant strains, which was divided into 18 sub-libraries (~100 strains each) (**S1 Fig**).

### Identification of EspF as a critical virulence protein for CR infection in *Il22*^-/-^ mice

**Fig 1:**
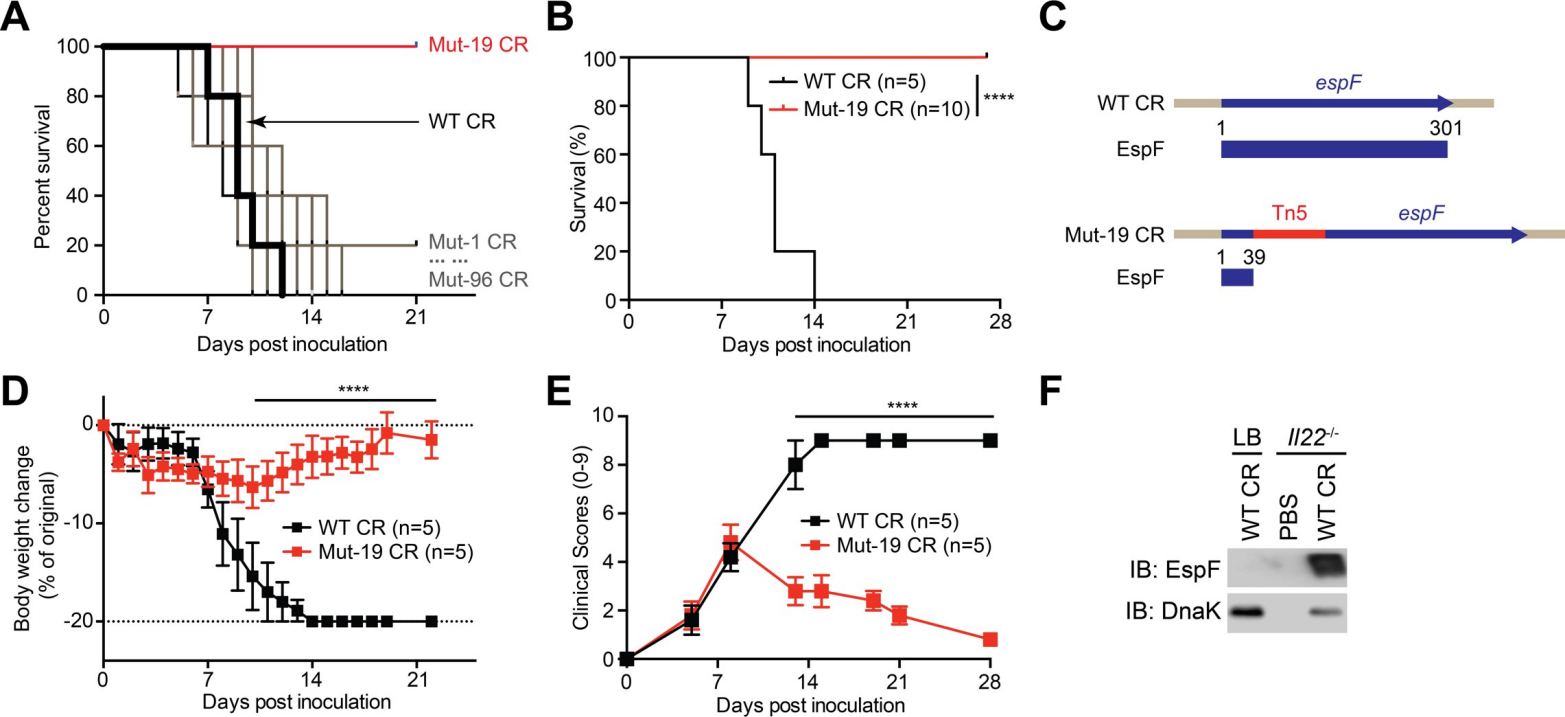
A functional screening reveals EspF as a novel virulence protein for CR virulence in *Il22*^-/-^ mice. **A.** Kaplan-Meier analysis of the survival rates in *Il22*^-/-^ mice inoculated with wild-type (WT) CR or each strain in the sub-library C consisting of 96 mutants (Mut-1 to Mut-96). **B.** Kaplan-Meier analysis of the survival rate in *Il22*^-/-^ mice inoculated with WT or Mut-19 CR. **C.** Schematics of normal or Tn5-interrupted *espF* genes and EspF protein expression in WT and Mut-19 CR, respectively. **D-E.** Weight loss (D) and clinical scores (E) of *Il22*^-/-^ mice at indicated days post inoculation (dpi) with WT or Mut-19 CR. **F.** *Il22*^-/-^ mice were inoculated with phosphate-buffered saline (PBS) or wild-type (WT) CR. At 7 dpi, WT CR was derived from the infected colon epithelia or LB culture and immunoblotted (IB) for EspF, with DnaK as a loading control. **** *p* < 0.0001 by Gehan-Breslow-Wilcoxon tests (B) and by Student’s *t* tests (D-E).

To explore the novel virulence protein(s) that is critical for CR infection-caused severe mortality and morbidity in *Il22*^*-/-*^ mice, we infected *Il22*^*-/-*^ mice by oral gavage with each individual mutant strain from the first 5 sub-libraries of CR random mutants (**S1 Fig**). Consistent with previous findings [14], wild-type CR infection in *Il22*^*-/-*^ mice led to 100% mortality within 14 days post inoculation (dpi) (**Figs 1A** and **S2A**). As expected, the mortality rates of *Il22*^*-/-*^ mice infected with most randomly mutated strains were comparable to that of animals with wild-type CR infection (**Figs 1A** and **S2A**). In striking contrast, infecting *Il22*^*-/-*^ mice with two mutant CR strains, *i*.*e*. Mut-19 in the sub-library C and Mut-61 in the sub-library D, failed to cause any mortality even at 28 dpi (**Figs 1A and 1B** and **S2A and S2B**). Tn5 transposon-targeted PCR followed by Sanger sequencing revealed that the Tn5 transposon was inserted into the *escN* gene coding the essential structural component EscN of the T3SS [3] in Mut-61 CR (**S2C Fig**) and the *espF* gene coding a T3SS effector EspF [39] in Mut-19 CR (**Fig 1C**), respectively. Indeed, the Tn5 transposon interruption caused major truncations in EscN and EspF proteins, which could substantially impair the proteins’ functions (**Figs 1C** and **S2C**). Consistent with the crucial role of EscN in T3SS function and CR colonization as shown in previous studies [3], the *escN*-interrupted Mut-61 CR failed to colonize in *Il22*^*-/-*^ mice at 7 dpi (**S2D Fig**). In contrast, the *espF*-interrupted Mut-19 CR exhibited no colonization defect in *Il22*^*-/-*^ mice (**S2D Fig**), suggesting that EspF could play an important role beyond colonization in CR pathogenesis.

In line with the severe mortality (**Fig 1A and 1B**), infection with wild-type CR caused more body weight loss and elevated clinical score (referring to both weight loss and diarrhea severity) in *Il22*^*-/-*^ mice within 7–14 dpi (**Fig 1D and 1E**). In contrast, animals infected with Mut-19 CR exhibited less weight loss, reduced diarrhea severity, and no mortality (**Fig 1B, 1D and 1E**). These results suggest that an intact *espF* gene could be crucial for CR infection-caused severe symptoms in *Il22*^*-/-*^ mice. Moreover, the levels of EspF were significantly elevated in CR derived from the infected *Il22*^*-/-*^ mice than those in the bacteria cultured in Luria–Bertani (LB) medium (**Fig 1F**), hinting that EspF could play an important function during CR infection *in vivo*. Together these results indicate that the T3SS effector protein EspF could be pivotal for CR-caused severe mortality and morbidity in *Il22*^*-/-*^ mice.

### Deletion of *espF* attenuates CR virulence in *Il22*^-/-^ mice

**Fig 2:**
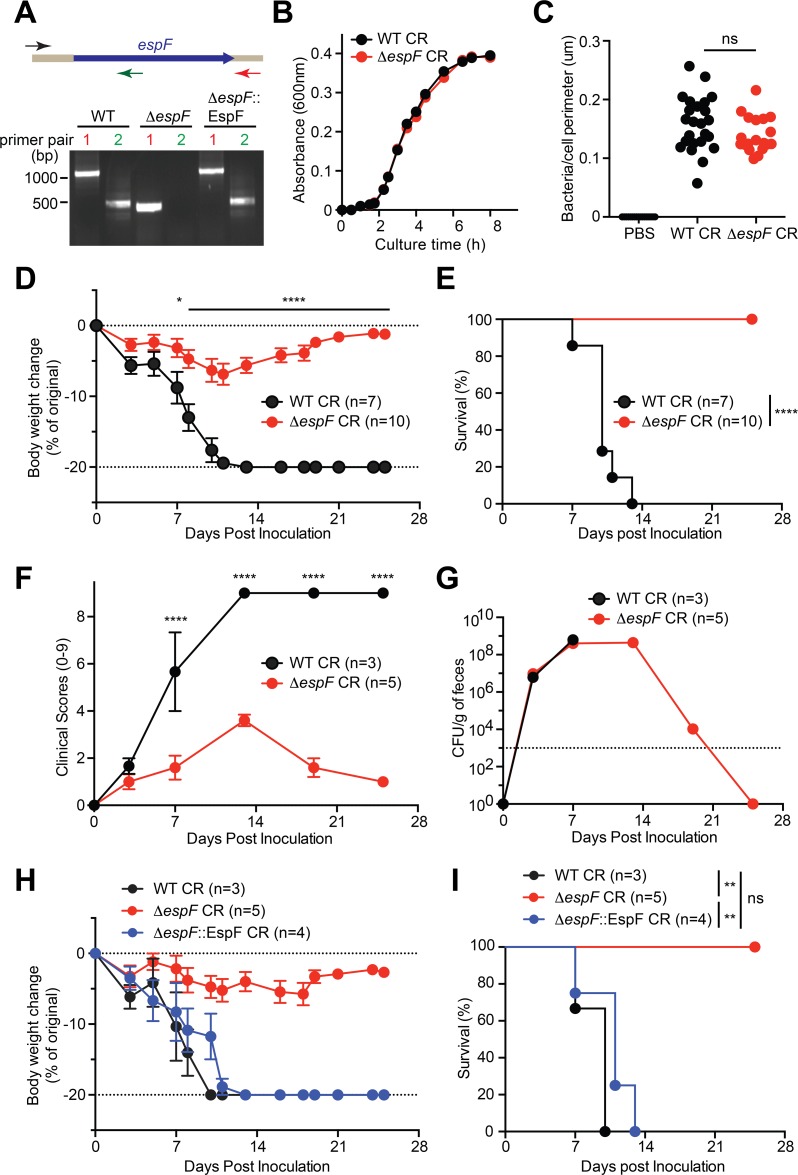
EspF is critical for CR infection-induced morbidity and mortality in *Il22*^-/-^ mice. **A.** Upper, diagram shows CR *espF* gene and PCR products amplified using a common forward primer and pair 1 reverse primer (in red), or pair 2 reverse primer (in green). Bottom, representative image of amplified PCR products using the indicated primer pairs from the CR strains as indicated. **B.** Growth curves of WT and *ΔespF* CR in LB medium, at 1: 150 dilutions from the overnight cultures. **C.** Colon epithelial cells (CEC) derived *Il22*^-/-^ mouse were infected in suspension with PBS, WT or *ΔespF* CR at 100 MOI for 3h, followed by immunofluorescence staining for CR-specific antisera, with nuclei counterstained by DAPI. The numbers of CR attached to CECs were quantified and normalized to the perimeter of individual CEC. **D, F.** Weight loss (D) and clinical scores (F) of *Il22*^-/-^ mice at indicated periods post inoculation with WT or *ΔespF* CR. **E.** Kaplan-Meier analysis of the survival rate in *Il22*^-/-^ mice inoculated with WT or *ΔespF* CR. **G.** Colonization kinetics of WT or *ΔespF* CR in *Il22*^-/-^ mice, inoculated with 2 × 10^9^ CFU of CR. **H-I.** Weight loss (H) and survival rate (I) of *Il22*^-/-^ mice inoculated with WT, *ΔespF*, or *ΔespF*::EspF CR. ns, not significant; * *p* < 0.05, ** *p* < 0.01, and **** *p* < 0.0001 by Student’s *t* tests (C-D, F), by Gehan-Breslow-Wilcoxon tests (E), and by Long-rank test with Bonferroni’s multiple comparison adjustment (I).

To rule out the possible off-target effect by Tn5 transposon insertion in Mut-19 CR, a *ΔespF* strain, in which the *espF* gene was chromosomally deleted [40] (**Fig 2A**), was utilized to ascertain our findings. We first monitored the growth dynamics of wild-type and *ΔespF* CR in LB culture, in order to examine whether EspF impacts bacterial proliferation. Indeed, *ΔespF* strain exhibited no defect in bacterial growth in LB media, compared to wild-type CR (**Fig 2B**). We then carried out *in vitro* infection assays to assess the capacities of wild-type and *ΔespF* CR strains to attach to primary mouse colon epithelial cells (CECs), and observed that wild-type and *ΔespF* CR attached to CECs in comparable numbers (**Fig 2C**). These results demonstrate that EspF is dispensable for CR colonization and proliferation *in vitro*.

To assess the impact of EspF on CR virulence, we infected *Il22*^*-/-*^ mice with wild-type and *ΔespF* CR strains. Consistently, infection with wild-type CR caused substantial weight loss, elevated clinical score, and severe lethality with 100% death in *Il22*^*-/-*^ mice within 14 dpi (**Fig 2D–2F**); whereas *ΔespF* CR infection resulted in substantially less weight loss, attenuated diarrhea severity and mortality in the infected animals (**Fig 2D–2F**), as did the Mut-19 CR strain (**Fig 1A–1D**). These results further underscore that EspF is critical for CR-caused severe mortality and morbidity in *Il22*^*-/-*^ mice. We further examined the colonization and proliferation capacities of wild-type CR and *ΔespF* strain in the infected *Il22*^*-/-*^ colon by counting viable CR recovered from the stool samples. The colony formation units (CFUs) of wild-type and *ΔespF* CR in feces derived from infected *Il22*^*-/-*^ mice at 3 and 7 dpi were almost identical (**Fig 2G**). Therefore, deletion of *espF* does not impact CR colonization and proliferation in the infected *Il22*^*-/-*^ mice *in vivo*, ruling out the possibility that EspF ablation alters bacterial burden to abolish the morbidity and mortality rates in infected animals.

To ascertain that EspF accounts for the attenuated mortality in *ΔespF* strain-infected *Il22*^*-/-*^ mice, we inoculated *Il22*^*-/-*^ animals with *ΔespF*::*espF* strain, in which the *ΔespF* strain was chromosomally complemented with a functional copy of *espF* (**Fig 2A**). Indeed, the complementation strain fully restored CR virulence in the infected *Il22*^*-/-*^ mice, as illustrated by the dramatic weight loss and 100% mortality rate, as the wild-type CR did (**Fig 2H and 2I**). Thus, our results further underscore the requirement of EspF for the CR infection-induced mortality and morbidity in *Il22*^*-/-*^ mice.

### The *ΔespF* strain still causes colonic inflammation in *Il22*^-/-^ mice

**Fig 3:**
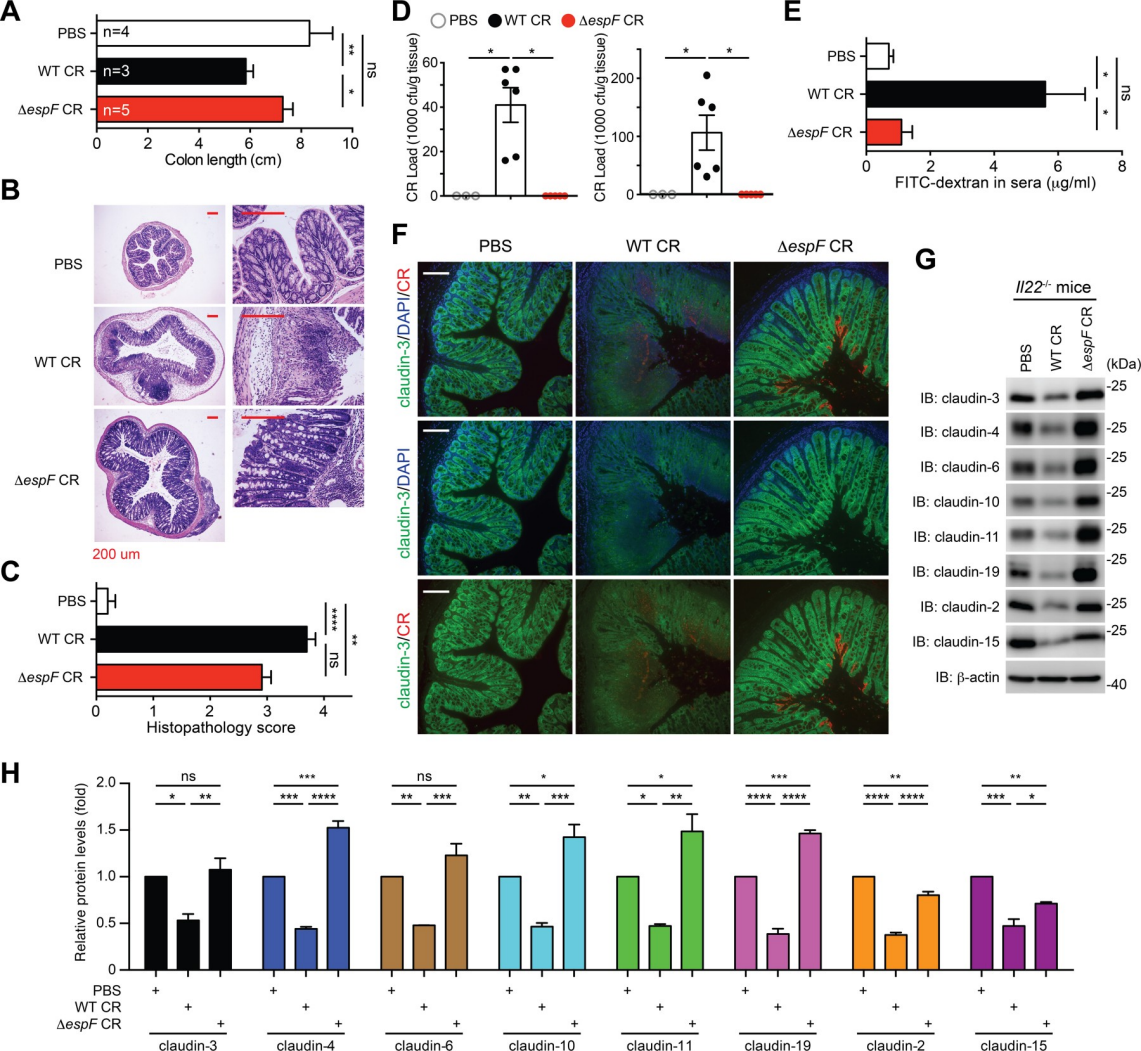
CR infection disrupts tight junction integrity in the colon in an EspF-dependent manner. **A.** Colon length of *Il22*^-/-^ mice, inoculated with phosphate-buffered saline (PBS) (n = 4), wild-type (WT, n = 3) or *ΔespF* (n = 5) CR, and euthanized at 7 days post inoculation (dpi). **B**. Hematoxylin and eosin staining of colons derived from *Il22*^-/-^ mice infected as in (A). Scale bars, 200 μm. **C.** The histopathology scores of colon sections derived from *Il22*^-/-^ mice infected as in (B). Shown are mean ± s.e.m of 10 random fields from two independent experiments. **D.** The CR burden in the liver (left) and the spleen (right) derived from *Il22*^-/-^ mice, infected as in (A) at 7 dpi, were quantified. **E.** Serum levels of FITC-dextran, assessed at 4 h post oral administration, in *Il22*^*-/-*^ mice infected with PBS (n = 5), WT CR (n = 9), or *ΔespF* CR (n = 4) at 7 dpi. **F.** Immunofluorescence micrographs of claudin-3 in the colons collected from *Il22*^-/-^ mice, infected as in (A) at 7 dpi, with nuclei counterstained by DAPI. Scale bars, 100 μm. **G.** *Il22*^-/-^ mice were infected as in (F), and colon epithelial cell lysates were derived and IB for indicated claudins, with β-actin as a loading control. **H.** The protein levels of indicated claudins, normalized to β-actin and PBS controls, were quantified by ImageJ software from three independent experiments. ns, not significant; * *p* < 0.05, ** *p* < 0.01, *** *p* < 0.001, and **** *p* < 0.0001 with one-way analysis of variance, followed by Bonferroni’s multiple comparison tests (A, D-E, H), and with Kruskal-Wallis test followed by Dunn’s multiple comparisons tests (C).

We sought to perform pathological analyses on the colonic tissues from *Il22*^*-/-*^ mice infected with PBS, wild-type CR, or *ΔespF* strain to assess the impact of EspF on the host responses at the epithelial surface. Indeed, wild-type CR infection, unlike the PBS control, caused discernible morphological damage to the infected colon, characterized by the shortening in colon length (**Fig 3A**). In contrast, no colon length shortening was observed in *Il22*^*-/-*^ animals infected with *ΔespF* CR or treated with PBS control (**Fig 3A**). Moreover, histological analyses revealed that wild-type CR infection caused severe damage to colon epithelial tissue in *Il22*^*-/-*^ mice, characterized by intestinal crypt elongation, goblet cell depletion, and immune cell infiltration (**Fig 3B and 3C**). Unexpectedly, *ΔespF* CR infection still resulted in substantial infiltration of immune cells and moderate tissue damage in the *Il22*^*-/-*^ colons, as illustrated by Hematoxylin and Eosin (H&E) staining-based histological analyses (**Fig 3B and 3C**). In line with the evidence that the *ΔespF* strain has almost identical colonization and proliferation rates as wild-type CR in infected *Il22*^*-/-*^ mice (**Fig 2G**), deletion of *espF* slightly attenuated CR induced inflammatory response and tissue damage to colon epithelia in *Il22*^*-/-*^ mice.

### EspF is essential for systemic CR spread in *Il22*^-/-^ mice

Given the observed severe tissue damage in the colon derived from wild-type CR-infected *Il22*^*-/-*^ mice (**Fig 3B and 3C**), we monitored bacterial load in the peripheral organs of infected animals at the peak of infection, to assess the impact of EspF on the translocation of CR through the damaged colonic epithelium. Indeed, CR burden in the liver and the spleen was not detectable in PBS control-inoculated animals; conversely they were markedly elevated in wild-type CR-infected *Il22*^*-/-*^ mice (**Fig 3D**). In striking contrast, the augmented CR burden in these peripheral organs was completely abolished in the mice infected with *ΔespF* strain (**Fig 3D**), which suggests that EspF is crucial for the systemic spread of CR during infection. Moreover, we orally administrated FITC-dextran to the infected *Il22*^*-/-*^ mice at the peak of infection and assessed colonic barrier function by measuring translocated FITC-dextran in serum. As expected, wild-type CR infection led to significantly elevated serum levels of FITC-dextran in *Il22*^*-/-*^ mice, indicating severe barrier dysfunction (**Fig 3E**). In contrast, the serum levels of FITC-dextran in PBS- and *ΔespF* CR–infected *Il22*^*-/-*^ mice remained at comparable low levels (**Fig 3E**). These results suggest that EspF plays a pivotal role for CR in increasing colonic epithelial permeability, thus accelerating the systemic spread of CR during infection in *Il22*^*-/-*^ mice.

### EspF is indispensable for CR infection-caused colon epithelial tight junction disruption

As previous studies showed that EspF was capable of damaging TJ transmembrane proteins [23,41], we first ascertained the impact of EspF on TJ integrity during CR infection *in vitro* using CMT-93 cells, a murine rectal carcinoma cell line that possesses differentiated epithelial characteristics including junctional complex and microvilli [42]. Indeed, at 3 h post infection, the protein levels of claudin-3, one of the most abundant TJ transmembrane proteins [43,44], were profoundly decreased in CMT-93 cells infected with wild-type CR, in comparison to PBS control (**S3 Fig**), indicating impaired claudin-3 dynamics or possible degradation, as previously shown in other types of cells [41]. Consistently, when *ΔespF* CR was employed, claudin-3 protein levels remained intact to a similar extent as those in mock-infected cells (**S3 Fig**). Our results confirmed that EspF is crucial for CR infection induced TJ disruption in CMT-93 cells *in vitro*.

We further examined whether CR infection triggers TJ disruption in the colon of infected *Il22*^*-/-*^ animals. As assayed by immunofluorescence staining, CR infection led to a clearly disorganized distribution of claudin-3 on colon tissue sections derived from *Il22*^*-/-*^ mice infected with wild-type CR, compared to PBS control, at 7 dpi (**Fig 3F**). Moreover, the mean fluorescence intensity of claudin-3 was substantially weakened in wild-type CR-infected colon sections, which indicates altered claudin-3 dynamics or degradation during CR infection *in vivo*, consistent with previous studies in immunocompetent animals [22,33]. In contrast, *ΔespF* CR infection failed to cause the disorganized distribution of claudin-3 on colon tissue sections (**Fig 3F**), although the attachment of *ΔespF* CR to colonic epithelium was identical to, if not higher than, that of wild-type CR. Indeed, as illustrated by immunoblot on the derived colonic epithelial lysates, wild-type CR infection caused striking decreases in the protein levels of occlusive claudins (*e*.*g*. claudin-3, -4, -6, -11, and -19) and pore-forming claudins (*e*.*g*. claudin-2 and -15), as well as some TJ-associated proteins and EspF-interacting proteins including ZO-1, 14-3-3σ, profilin, and arp2 in the infected *Il22*^*-/-*^ mice (**Figs 3G and 3H** and **S4**). In contrast to WT CR-infected animals, the levels of colonic claudin-3, -4, -6, -10, -11, and -19 derived from *ΔespF* CR infected mice remained comparable to, or even higher than, those in PBS-inoculated controls (**Fig 3G and 3H**). Interestingly, EspF deletion in CR led to smaller impacts on pore-forming claudin-2 and claudin-15 than on the occlusive claudins (**Fig 3G and 3H**), which is indicative of an EspF-mediated differential impact on these two important classes of claudins in TJs.

To examine whether EspF is crucial for other activities (such as bloodstream survival, immune evasion, and extra-intestinal colonization) during CR systemic dissemination, we infected *Il22*^*-/-*^ mice with a mixed inoculum that contained WT CR and *ΔespF* CR at a 1:1 ratio. As expected, the WT-*ΔespF* CR coinfection in *Il22*^*-/-*^ mice led to severe body weight loss, 100% lethality, and systemic dissemination of CR in the liver and the spleen (**S5A–S5C Fig**), as WT CR alone did (**Figs 2D and 2E** and **3D**). Our PCR-based identification verified the WT-*ΔespF* ratio (47% versus 53%) in the mixed inoculum (**S5D Fig**). Interestingly, the WT-*ΔespF* ratios in the CR derived from the liver and the spleen of the mixed CR-infected *Il22*^*-/-*^ mice at 7 dpi remained comparable to that at 0 dpi (**S5D Fig**), indicating that *ΔespF* CR does not possess competitive disadvantage in systemic dissemination when colonic barrier is disrupted. *ΔespF* CR exhibited similar capability of disseminating from the colon to the peripheral organs compared to WT CR, which suggests that EspF appears dispensable for bloodstream survival, immune evasion, and extra-intestinal colonization during CR systemic dissemination. Hence, these results, in line with the systemic spread of CR (**Fig 3D**) and augmented epithelial membrane permeability (**Fig 3E**), suggest that EspF is critical for CR-induced TJ disruption in the colon of *Il22*^*-/-*^ mice.

### The N-terminal 119 residues of EspF is sufficient for CR-induced lethality in *Il22*^-/-^ mice

EspF is conserved among A/E pathogens with a highly conserved N-terminal region and a C-terminal region consists of various eukaryotic-like proline-rich repeats (PRRs). EPEC-, EHEC-, and CR-EspF harbor 3, 4, and 5 copies of PRRs, respectively (**S6A Fig**), whereas the pathophysiological relevance of these PRRs has not been extensively investigated yet. To explore the possible role of PRRs within EspF in CR-caused lethal colitis in *Il22*^*-/-*^ mice, we generated full-length and truncated CR-EspF constructs in pACYC184 plasmid (**S6B Fig**). Among the constructs, full-length and truncated PRR1, PRR2, and PRR3 were ectopically expressed in *ΔespF* CR; whereas the expression of PRR0 (only N-terminus without any PRR) and PRR4 (containing the first 4 PRRs) was not detectable, most likely due to the instability of the truncated proteins (**S6C Fig**). Indeed, the plasmid-complemented *ΔespF*/pEspF-Flag CR restored CR virulence in the infected *Il22*^*-/-*^ mice, assayed by body weight loss and lethality (**S6D and S6E Fig**), as the chromosomally complemented *ΔespF*::*espF* strain and wild-type CR did (**Figs 2H and 2I** and **S6D and S6E**). Interestingly, *ΔespF* strains that express PRR1, PRR2, or PRR3 all led to more severe body weight loss and quicker mortality in *Il22*^*-/-*^ mice, compared to *ΔespF*/pEspF-Flag CR (**S6D and S6E Fig**), which suggests that the N-terminus and PRR1 within EspF are sufficient to execute its pathogenic function in CR-induced lethality in *Il22*^*-/-*^ mice. Of note, a conserved functional mitochondrial targeting signal (MTS) within the N-terminus of EspF was identified previously and substitution of the 16^th^ leucine with glutamic acid (L16E) in EspF completely abolished its mitochondrial localization [45]. To examine whether the N-terminal MTS, particularly the leucine-16 of EspF plays an important role in CR-induced lethality in *Il22*^*-/-*^ mice, we generated *ΔespF*/pEspF(L16E)-Flag CR (**S7A Fig**) and confirmed that EspF-Flag proteins were expressed at comparable levels in *ΔespF*/pEspF-Flag CR and *ΔespF*/pEspF(L16E)-Flag CR (**S7B Fig**). Infecting *Il22*^*-/-*^ mice with *ΔespF*/pEspF(L16E)-Flag CR or *ΔespF*/pEspF-Flag CR led to severe body weight loss and 100% mortality rate at 16 dpi (**S7C and S7D Fig**), indicating that N-terminal 16^th^ leucine and MTS are not critical for EspF function in CR-caused lethality in *Il22*^*-/-*^ mice. Together our results support the essential role of N-terminal 119 amino acids containing the PRR1 in EspF-mediated CR pathogenesis in *Il22*^*-/-*^ mice.

### Tight junction-disrupting virulence genes are upregulated during CR infection

**Fig 4:**
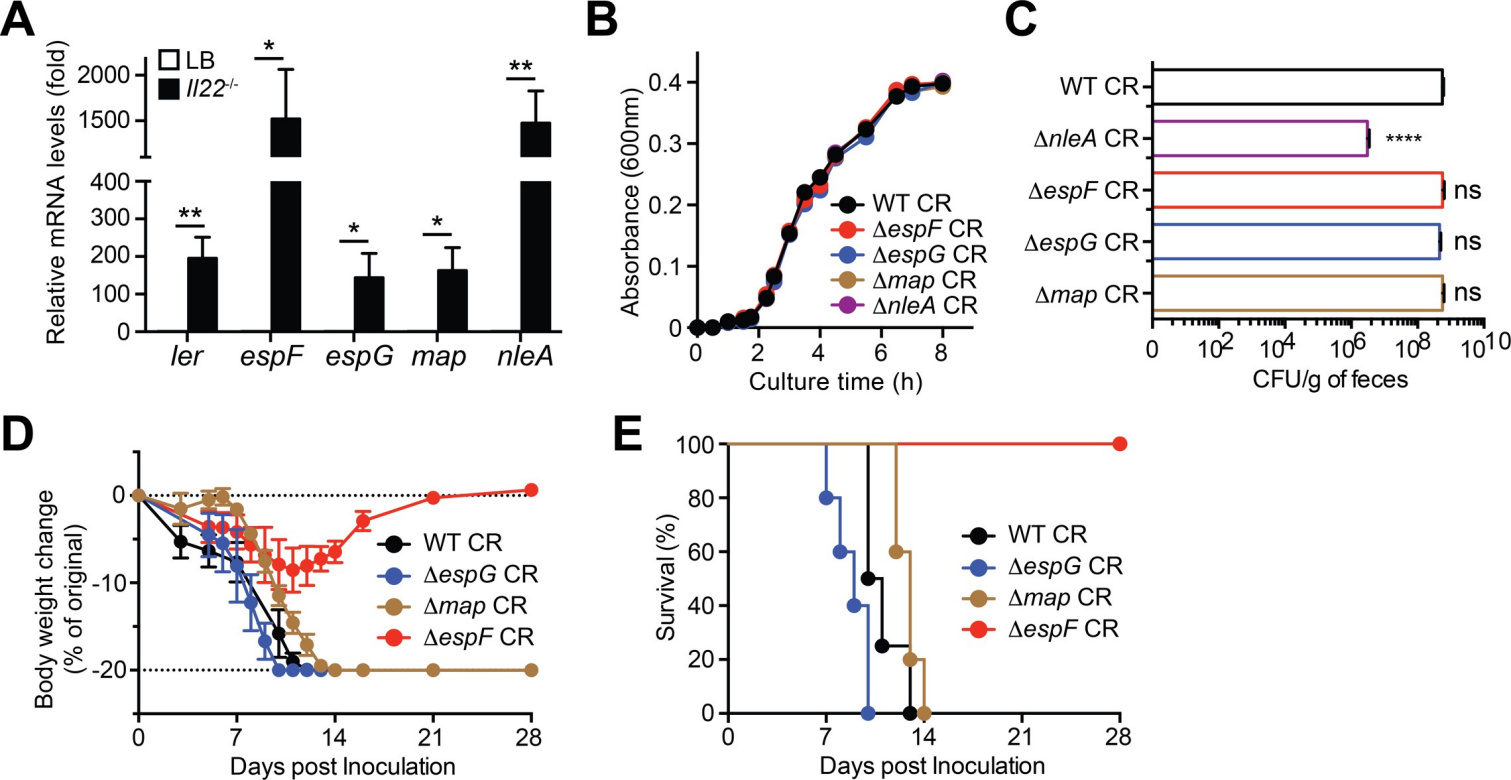
EspF plays a specific role for CR infection-caused lethality in *Il22*^-/-^ mice. **A.** Quantitative PCR was used to determine mRNA levels of *ler*, *espF*, *espG*, *map*, and *nleA* relative to 16S rRNA in the CR collected from LB culture or *Il22*^-/-^ mice at 7 days post inoculation (dpi) with wild-type *Citrobacter rodentium* (CR). Shown are mean ± SEM from 5 inoculated *Il22*^-/-^ mice, with mRNA levels in CR derived from LB culture set a 1. **B.** Growth curves of indicated CR strains in LB medium, at 1:150 dilutions from the overnight cultures. **C.** The colony formation units (CFU) of live CR derived from fecal samples of *Il22*^-/-^ mice, inoculated with 2 × 10^9^ CFU of indicated CR strains, at 7 dpi. **D-E.** Weight loss (D) and survival rate (E) of *Il22*^-/-^ mice inoculated with WT (n = 4), *ΔespG* (n = 5), *Δmap* (n = 5), or *ΔespF* (n = 5) CR. ns, not significant, * *p* < 0.05, ** *p* < 0.01, and **** *p* < 0.001 by Student’s *t* tests (A) and with one-way analysis of variance, followed by Bonferroni’s multiple comparison tests (C).

We further carried out qRT-PCR assays to examine the transcriptional regulation of known virulence genes disrupting TJ dynamics during CR infection in *Il22*^*-/-*^ mice. Indeed, the messenger RNA (mRNA) levels of *Ler*, an essential virulence regulatory gene [46], in wild-type CR derived from the infected *Il22*^*-/-*^ mice at 7 dpi were dramatically elevated compared to those in CR from LB culture (**Fig 4A**), which supports that CR undergoes substantial transcriptional rearrangement when adapting to the host [3]. In line with the elevated EspF protein levels in CR derived from the infected *Il22*^*-/-*^ mice (**Fig 1F**), the mRNA levels of *espF* were markedly higher in the host-derived CR compared to those in LB-cultured bacterium (**Fig 4A**), as did those of *espG*, *map*, and *nleA* encoding other known TJ-damaging virulence proteins (**Fig 4A**). Hence these results suggest that many virulence genes encoding TJ-disrupting effectors are upregulated in general, in line with the severe damage to colonic epithelial barrier integrity (**Fig 3D**), during CR infection in *Il22*^*-/-*^ animals.

### A specific role of EspF for CR infection-induced virulence in *Il22*^-/-^ mice

To examine whether the known TJ-disrupting effectors EspF, EspG, Map, and NleA execute redundant functions at colonic epithelia during CR infection *in vivo*, we assessed their relevance in CR infection-caused mortality in *Il22*^*-/-*^ mice. When cultured in LB medium *in vitro*, *ΔespG*, *Δmap*, and *ΔnleA* strains harbored the same proliferation dynamics as wild-type and *ΔespF* CR (**Fig 4B**), suggesting that none of these genes are essential for bacterial growth. To assess their effects on the CR burden during infections *in vivo*, we inoculated them into *Il22*^*-/-*^ animals. Our analyses showed that the fecal CFUs of wild-type, *ΔespF*, *ΔespG*, and *Δmap* CR derived from the infected mice at 7 dpi were largely comparable (**Fig 4C**), indicative of intact colonization and proliferation of CR in the host. In contrast, the fecal CFUs of *ΔnleA* CR were dramatically attenuated compared to other strains (**Fig 4C**), indicating a marked defect in CR colonization and proliferation *in vivo*. We therefore excluded the *ΔnleA* strain from infection-induced lethality in *Il22*^*-/-*^ mice. Consistent with our above findings (**Fig 2D and 2E**), infection with *ΔespF* CR caused minor body weight loss and no death in *Il22*^*-/-*^ mice, even after 21–28 dpi (**Fig 4D and 4E**). In striking contrast, despite similar colonization and proliferation, infections with *ΔespG* and *Δmap* strains led to remarkable body weight loss, diarrheal symptoms, and severe mortality with 100% death within 14 dpi in *Il22*^*-/-*^ mice, as did wild-type CR (**Fig 4D and 4E**). These results suggest that EspF plays a specific role, rather than a redundant function to other TJ-disrupting effectors EspG and Map, in CR infection-caused severe lethality in *Il22*^*-/-*^ mice.

## Discussion

Gastrointestinal infections caused by A/E pathogens EPEC and EHEC, which are associated with diarrheal diseases, impose significant economic burdens and remain a major public health issue [1,2,3]. Understanding the pathogenic mechanisms of EPEC/EHEC will greatly facilitate new discoveries for treatment against EPEC/EHEC infections in humans. Unfortunately, EPEC and EHEC do not infect mice well under specific pathogen free conditions, which substantially impedes disease modeling and discoveries of pathogenic mechanisms, especially the host-pathogen interactions in pathophysiological settings. The majority of the pathogen-encoded genes and pathogenic strategies are shared among EPEC, EHEC, and their murine equivalent CR, which makes CR infection in mice a widely used small animal model to investigate the EPEC/EHEC pathogenicity, although a substantial amount of pathophysiological pathogen-host interactions are still elusive [3]. A/E pathogens are known to cause a self-limited infection with transient inflammation and mild symptoms in immunocompetent hosts [3]. A recent study demonstrates that CR infection triggers a strikingly elevated mortality in *Il22*^*-/-*^ mice, but not wild-type C57Bl/6 animals [14]. Moreover, the Global Enteric Multicenter Study reveals that EPEC is a leading cause of lethality associated with diarrhea among children less than 12 months of age [20,23,39]. The low circulating IL-22 levels in human neonates was speculated to be a significant risk factor for serious infections in the neonatal period [47]. These mouse model and epidemiological studies underscore the severe symptoms and outcomes of A/E pathogen infections in immunocompromised and/or immunodeficient hosts. However, studies of A/E pathogens in immunocompromised and/or immunodeficient settings significantly lag behind those of immunocompetent settings. Hence there is an urgent need to identify the crucial bacterial virulence proteins in A/E pathogens that are important for the infection-associated severe symptoms in the immunocompromised conditions.

CR infection-caused striking phenotypes, *i*.*e*. severe mortality, in *Il22*^*-/-*^ mice and the well-established genetic manipulation in CR allowed us to systematically explore novel virulence protein(s) critical for CR infection-associated virulence in the immunocompromised host and identify EspF as a key effector under pathophysiological condition. EspF, located on the fourth operon of *LEE* pathogenicity island, was originally identified based on its similarity in amino acid sequence, *i*.*e*. the proline-rich repeats, to eukaryotic motifs [39]. Such eukaryotic motifs allow EspF to interact with many host proteins after injection via the T3SS, thus manipulating diverse cellular processes, in particular disruption of the epithelial barrier [48]. The first finding that relates EspF to epithelial barrier disruption was an observation made in EPEC infection on T84 monolayers [23]. While EPEC produces a disorganized distribution of occludin, a major TJ transmembrane protein, leading to progressive loss of TER [23,24], an *espF* mutant was deficient in distributing occludin from the TJs and disrupting TER of T84 monolayers [23]. Moreover, EspF was proposed to interact with a variety of proteins involving in actin nucleation and TJ dynamics such as sorting nexin 9 (SNX9), SNX33, N-WASP, profilin, ZO-1, ZO-2, and actin [29,30,31,49]; however, the precise mechanism on EspF-mediated TJ protein redistribution is yet to be elucidated [20]. It is noteworthy that while EspF has been extensively investigated using *in silico* analyses, yeast double hybrid assays, recombinant proteins, and *in vitro* infection models [29,30,31,49], only few studies explored the relevance of EspF during A/E pathogen infections in immunocompetent hosts *in vivo* [22,28,32]. Our results demonstrate that EspF is transcriptionally regulated in CR at the host-pathogen interface during infection *in vivo*. More importantly, our findings reveal that EspF is an indispensable effector crucial for CR infection-induced severe mortality in animals under immunocompromised conditions, thus suggesting another important and pathophysiological function to this multifunctional bacterial effector that is conserved among A/E pathogens.

TJs in the colon epithelium are critical for preventing the entry of pathogens as well as enteric flora during A/E pathogen infections [20]. In opposition, A/E pathogens inject various T3SS effectors into the host cell, where they manipulate the signaling pathways associated with TJ dynamics [20]. The T3SS effectors EspF, EspG, Map, and NleA were previously reported to be critical for A/E pathogen-induced decrease in TER when different types of cells cultured in monolayer were infected [23,24,25,26,27]. Of note, these effectors interact with distinct proteins and disturb different TJ transmembrane proteins and cytosolic scaffold proteins in the host cells, ranging from claudins, occludin, ZO-1/2 to actin [20]. However, the exact transmembrane and scaffold proteins within the complex TJ structure disrupted by EspF, EspG, Map, and NleA have not been fully understood. Mediated by their distinct multifunctional nature, these effectors are speculated to independently disrupt TJ strands through distinct mechanisms [50] or function in a coordinated manner [20]. That said, the relevance of all these effectors have not been investigated in the same pathophysiological setting. Intriguingly, deletion of *espF* alone, but not *espG* or *map*, highly attenuates CR virulence in *Il22*^*-/-*^ mice, suggesting that they do not function in a redundant or synergistic relationship under this specific condition. Of note, EspF deficiency does not abolish the CR infection-initiated inflammatory response and colonic tissue damage, consistent with the dispensable role of EspF in forming A/E lesions in T84 intestinal epithelial cells by EPEC *in vitro* [23]. However, deletion of *espF* completely abolishes the CR infection-dependent TJ disruption, epithelial barrier loss, and severe lethality in *Il22*^*-/-*^ animals. In summary, we reveal that EspF-targeted TJ disruption is pivotal for maintaining epithelial permeability and coordinating mucosal immune activation during CR infection under immunocompromised conditions.

## Materials and methods

### Ethics statement

All animal experiments were performed according to protocol number MO16H285, approved by the Johns Hopkins University’s Animal Care and Use Committee and in direct accordance with the NIH guidelines for housing and care of laboratory animals. *Il22*^-/-^ (IL-22 knockout) mice in C57Bl/6 background [47] were maintained in a specific pathogen-free facility and fed autoclaved food and water *ad libitum*.

### Cell culture, antibodies, reagents, and plasmids

CMT-93 cells (ATCC, Manassas, VA) and mouse CECs isolated as previously described [51], were cultured in DMEM medium containing 10% fetal calf serum, 2 M glutamine, 100 U ml^-1^ penicillin, and 100 U ml^-1^ streptomycin. Antibodies used were: DnaK (8E2/2, 50667656), claudin-3 (34–1700), claudin-15 (38–9200), ZO-1 (40–2300) from Thermo Fisher Scientific (Halethorpe, MD); claudin-4 (A12, sc-376643), claudin-6 (A-4, sc-393671), claudin-10 (G-12, sc-373946), claudin-11 (D-8, sc-271232), claudin-19 (C-5, sc-365967), Arp2 (E-2, sc-137250), Profilin (B-10, sc-137235), 14-3-3σ (E-11, sc-166473) from Santa Cruz Biotechnology (Dallas, TX); β-actin (AC-15, A5441) and Flag (M2, F3165) from Sigma-Aldrich (St. Louis, MO); claudin-2 (ab53032) from Abcam (Cambridge, MA); EspF antiserum was described previously [39]; and polyclonal antiserum specific for *Citrobacter rodentium* was generated using heat-killed CR as an immunogen following a standard immunization protocol. 4',6-diamidino-2-phenylindole (DAPI) were obtained from Sigma-Aldrich. The full length and truncated EspF-Flag were amplified from wild-type *C*. *rodentium* genomic DNA using the primers detailed in S2 Table, and cloned into pACYC184 plasmid using Gibson Assembly Master Mix (New England BioLabs, Ipswich, MA). The EspF(L16E) mutant was generated by site-directed mutagenesis using the Quick Change Kit (NEB) with appropriate primers for L16E. All constructs were verified by Sanger sequencing.

### Random mutagenesis in Citrobacter rodentium

Wild-type *C*. *rodentium* (DBS 100 strain) [51] was randomly mutagenized by Tn5 transposon insertion, using an EZ-Tn5-KAN-2 TNP Transposome Kit (Illumina, San Diego, CA), per the manufacturer's instructions. Briefly, 20 ng of EZ-Tn5 Transposase enzyme and the EZ-Tn5 <KAN-2> Transposon were electroporated using a Bio-Rad MicroPulser (Bio-Rad Laboratories, Hercules, CA) using program Ec1 with pulse controller set at 1.8 kV into electrocompetent wild-type *C*. *rodentium* where the EZ-Tn5 transposase was activated by Mg^2+^ in the host’s cellular environment resulting in random insertion of the EZ-Tn5 Transposon into *C*. *rodentium* genomic DNA. The Tn5-interrupted *C*. *rodentium* mutants were recovered in pre-warmed SOC medium, plated onto LB agar plates containing 25 μg/ml of kanamycin, and incubated at 37°C for 18 h. All colonies were picked and propagated in 96-well plates in LB medium containing 50 μg/ml of kanamycin. Arbitrary PCR followed by Sanger sequencing was carried out to determine the transposon insertion site in *C*. *rodentium* genome.

### *Citrobacter rodentium* culture

Wild-type, *ΔespF*, *ΔespG*, *ΔnleA*, *Δmap* strains of *C*. *rodentium* were previously described [34,40,52], as detailed in S1 Table. The *ΔespF*::EspF was generated using the “scar”-free in-frame deletion method using the suicide vector pRE112 via *sacB*-based allelic exchange [53], with the primers detailed in S2 Table. The *C*. *rodentium* strains were grown from single colonies on LB plates in LB broth at 37°C overnight with shaking. For growth curve measurement, the overnight *C*. *rodentium* culture was diluted at 1: 200 and grown at 37°C with shaking, and 100 μL of *C*. *rodentium* culture was taken at indicated time periods to read OD_600_ on a 96-well plate using a POLARStar Omega Plate Reader (BMG Labtech, Cary, NC).

### *Citrobacter rodentium* infection *in vitro*

Infection of CR in CMT-93 and mouse CECs was performed as previously described [51]. Briefly, CR was washed with ice-cold PBS and resuspended in pre-warmed corresponding media. Bacteria concentration was measured by absorbance at optical density 600, followed by a serial dilution and seeding on a MacConkey agar plate to confirm the administered colony-forming units (CFU). The CMT-93 cells (grown as a monolayer) and isolated CECs (in suspension) were infected with CR at a multiplicity of infection (MOI) of 100 for 3 h, followed by whole cell lysis and immunoblotting or immunofluorescence staining. CR attachment to CECs was determined as the numbers of CR that attached to individual CEC, as previously described [51].

### *Citrobacter rodentium* infection in mice

Male *Il22*^-/-^ mice (8 to 16 weeks) were fasten for 8 h before orally inoculated with 200 μl of PBS containing 2 × 10^9^ CFU of indicated CR strains or PBS alone. Mice were observed daily for weight and morbidity, and the disease clinical scores were calculated as the sum of weight loss and diarrhea, using the following criteria as previously described [19]: weight loss (0 point = none, 1 point = 1%-5% weight loss, 2 points = 5%-10% weight loss, 3 points = 10%-15% weight loss, 4 points = more than 15~20% weight loss and 5 points = more than 20% weight loss); stool consistency/diarrhea (0 points = normal, 2 points = diarrhea (Feces consisting of well-formed pellets will be recorded as normal, whereas yellowish mud-like feces will be recorded as diarrhea), 4 points = watery diarrhea.

### Bacterial counts

For fecal CR burden analysis, stool was collected from live animals at various times post inoculation; for CR dissemination analysis, the liver and the spleen were collected from euthanized mice at 7 days post inoculation. The stool and tissue were homogenized, and diluted in PBS, and plated on MacConkey agar plates, incubated overnight at 37°C. CR CFUs were enumerated the following day and normalized to the stool or tissue weight.

### FITC-dextran assays

FITC-dextran assays for intestinal permeability were conducted as previously described [54]. Briefly, mice were orally administrated with 150 μL of 80 mg/mL FITC-dextran (4,000 Da, FD4, Sigma) in PBS. After 4 h, mice were anaesthetized and blood was collected by cardiac punctures. To deter coagulation, blood samples were immediately added to a final concentration of 3% acid-citrate dextrose (20 mM citric acid, 100 nM sodium citrate, and 5 mM dextrose) and centrifuged 1,000 × *g* at 4°C for 12 min. Plasma was collected for fluorescence reading using a BioTek Synergy HT microplate reader (BioTek, Winooski, VT) at excitation 485/20 nm and emission 528/20 nm.

### Colon tissue collection, histology, and immunofluorescence

Histology and immunofluorescence staining of colon tissue sections were performed as previously described [55]. In brief, after euthanizing mice, the colons were removed under aseptic conditions, the terminal 0.5-cm piece of the colon was fixed in 10% buffered formalin for 24 h, embedded in paraffin and 5-micron sections were cut and processed for Hematoxylin and Eosin (H&E) staining. Histopathology scores were determined using the following criteria as previously described [51]. For immunofluorescence staining, the colon tissue sections were subjected to antigen retrieval in Citrate buffer (pH 6.0), washed with PBS, and blocked with appropriate sera in PBS. After incubating with appropriate antibodies, sections were washed and incubated with fluorescence dye-conjugated second antibodies and 1 μg/ml of DAPI. Stained sections were washed and mounted under a coverslip using Fluoro-gel with Tris Buffer (Electron Microscopy Sciences, Hatfield, PA) and examined on a Leica DMi8 fluorescence microscope (Leica Microsystems, Wetzlar, Germany).

### Immunoblot

Immunoblot assay was conducted as previously described [51]. In brief, cells or colon tissues were harvested and lysed on ice by 0.4 ml of lysis buffer (50 mM Tris-HCl [pH 8.0], 150 mM NaCl, 1% NP-40 and 0.5% sodium deoxycholate, 1 × complete protease inhibitor cocktail [Roche Applied Science, Indianapolis, IN]) for 30 min. The lysates were centrifuged at 10,000 × *g* at 4°C for 10 min, followed by a separation by SDS-PAGE under reduced and denaturing conditions. The resolved protein bands were transferred onto nitrocellulose membranes and probed by the Super Signaling system (Thermo Fisher Scientific) according to the manufacturer's instructions, and imaged using a FluorChem E System (Protein Simple, Santa Clara, CA).

### Quantitative real-time PCR

After euthanizing mice, the colons were removed under aseptic conditions. 5 cm of colon were dissected longitudinally with lumen facing up, and the colon luminal contents consisting of colonized bacteria were scraped and suspended in bacterial enhancement reagent (Life Technologies). Bacterial total RNA was isolated using Trizol reagent (Life Technologies), followed by the elimination of genomic DNA using TURBO DNA-free Kit (Life Technologies). Complementary DNA (cDNA) synthesis was performed using qScript cDNA SuperMix Kit (Quanta Biosciences, Gaithersburg, MD). Target genes were amplified using SsoAdvanced SYBR Green Supermix (Bio-Rad Laboratories) with primers listed in S3 Table.

### Statistical analysis

All statistical analysis was performed using GraphPad Prism version 6.0 (GraphPad Software, San Diego, CA). Standard errors of means (s.e.m.) were plotted in graphs. Multiple comparison tests (either using One-way analysis of variance or Kruskal-Wallis depending on data distribution) followed by post-hoc tests (either Bonferroni’s or Dunn’s multiple comparisons tests) were detailed in the figure legends. Statistical analysis on survival curves was performed using the log-rank (Mantel-Cox) test and Bonferroni corrections were applied for multiple comparisons. Significant differences were considered: ns, non-significant difference; * at *p* < 0.05; ** at *p* < 0.01; *** at *p* < 0.001; **** at *p* < 0.0001.

## Supporting information

S1 Table *Citrobacter rodentium* (CR) strains used in this study. (XLSX)

S2 Table PCR primers used in this study. (XLSX)

S3 Table Quantitative real-time PCR primers used in this study. (XLSX)

S1 Fig Schematics of functional screen of a Tn5-interrupted CR mutant library in *Il22*^-/-^ mice. Wild-type CR transfected with EZ-Tn5-KAN-2 Transposome were recovered in SOC medium and plated onto kanamycin^+^ LB agar plates. All colonies (~2,000 strains) were picked and propagated in 96-well plates in LB medium containing 50 μg/ml of kanamycin. After removing the mutants with obvious growth defects, approximate 1,800 Tn5 mutant strains were divided into 18 sub-libraries (~100 strains each). The CR mutants from the first 5 sub-libraries (A-E) and wild-type CR were cultured overnight, and *Il22*^-/-^ mice (3–5 animals for each individual strain) were infected by oral gavage with 2 × 10^9^ CFU of each individual strain (~500 strains). The clinical symptoms and survival of *Il22*^-/-^ mice were monitored post infection for 28 days.
(PDF)

S2 Fig A functional screening reveals EscN as a key virulence protein for CR virulence in *Il22*^-/-^ mice. **A.** Kaplan-Meier analysis of the survival rates in *Il22*^-/-^ mice inoculated with wild-type (WT) CR or each strain in the sub-library D consisting of 96 mutants (Mut-1 to Mut-96). **B.** Kaplan-Meier analysis of the survival rate in *Il22*^-/-^ mice inoculated with WT or Mut-61 CR. **C.** Schematics of normal or Tn5-interrupted *escN* genes and EscN protein expression in WT and Mut-61 CR, respectively. **D.** The colony formation units (CFU) of live CR derived from fecal samples of *Il22*^-/-^ mice, inoculated with 2 × 10^9^ CFU of indicated CR strains, at 7 dpi. ns, not significant, **** *p* < 0.001 by Long-rank test (B) and with one-way analysis of variance, followed by Bonferroni’s multiple comparison tests (D).
(PDF)

S3 Fig CR infection disrupts tight junction in cultured CMT-93 cells in an EspF-dependent manner. **A.** Representative immunofluorescence micrographs of CMT-93 cells infected in suspension with PBS, WT or *ΔespF* CR at 100 MOI for 3h, with nuclei counterstained by DAPI. Scale bars, 20 μm. **B.** CMT-93 cells were infected as in (A) and whole cell lysates were derived and immunoblotted (IB) for claudin-3, with β-actin as a loading control.
(PDF)

S4 Fig EspF affects some TJ component and associated proteins during CR infection in *Il22*^-/-^ mice. **A.** *Il22*^-/-^ mice, inoculated with phosphate-buffered saline (PBS), wild-type (WT) CR, or *ΔespF* CR, and euthanized at 7 days post inoculation (dpi). Colon epithelial cell lysates were derived and immunoblotted (IB) for indicated proteins, with β-actin as a loading control. **B.** The indicated protein levels, normalized to β-actin and PBS controls, were quantified by ImageJ software from three independent experiments. * *p* < 0.05, ** *p* < 0.01, *** *p* < 0.001, and **** *p* < 0.0001 with one-way analysis of variance, followed by Bonferroni’s multiple comparison tests.
(PDF)

S5 Fig EspF is dispensable for the bloodstream survival, immune evasion, extra-intestinal colonization of CR during lethal infection in *Il22*^-/-^ mice. **A.** Weight loss of *Il22*^-/-^ mice at indicated periods post inoculation with 2 × 10^9^ CFU of mixed CR, with a ratio of wild-type (WT) CR to *ΔespF* CR at 1:1. **B.** Kaplan-Meier analysis of the survival rate in *Il22*^-/-^ mice inoculated with mixed WT CR and *ΔespF* CR as in (A). **C.** The CR burden in the liver and the spleen derived from *Il22*^-/-^ mice, infected as in (A) at 7 days post infection (dpi), were quantified. **D.** Live CR colonies were derived from the mixed inoculum at 0 dpi, or the liver and the spleen of infected *Il22*^-/-^ mice at 7dpi. Individual CR colony was pickup and subjected to PCR-based identification as WT CR or *ΔespF* CR (as illustrated in Fig 2A). Shown are percentages of WT CR and *ΔespF* CR in the indicated numbers of live CR colonies examined.
(PDF)

S6 Fig The N-terminus and PRR1 of EspF are required for CR infection-induced morbidity and mortality in *Il22*^-/-^ mice. **A.** Schematic diagram of the EspF proteins from EPEC, EHEC, and CR. PRR, proline-rich repeat. **B.** Schematic diagram of full-length CR-EspF and indicated truncated CR-EspF containing different PRRs, fused with a C-terminal Flag tag. **C.** Whole cell lysates derived from the indicated CR strains were immunoblotted (IB) for Flag, with DnaK as a loading control. The full-length and truncated EspF-Flag proteins are marked with arrows. **D.** Weight loss of *Il22*^-/-^ mice at indicated periods post inoculation with the indicated CR strains. **E.** Kaplan-Meier analysis of the survival rate in *Il22*^-/-^ mice inoculated with the indicated CR strains.
(PDF)

S7 Fig The N-terminal 16^th^ leucin of EspF is dispensable for CR-induced morbidity and mortality in *Il22*^-/-^ mice. **A.** Schematic diagram of full-length and L16E mutant CR-EspF, fused with a C-terminal Flag tag. PRR, proline-rich repeat. **B.** Whole cell lysates derived from the indicated CR strains were immunoblotted (IB) for Flag, with DnaK as a loading control. **C.** Weight loss of *Il22*^-/-^ mice at indicated periods post inoculation with the indicated CR strains. **E.** Kaplan-Meier analysis of the survival rate in *Il22*^-/-^ mice inoculated with the indicated CR strains.
(PDF)
