# Supplementary figures and images for: EspF is crucial for Citrobacter rodentium-induced tight junction disruption and lethality in immunocompromised animals
Source: PLoS Pathog. 2019 Jun 28;15(6):e1007898. doi: 10.1371/journal.ppat.1007898 (PMC6623547; doi:10.1371/journal.ppat.1007898)

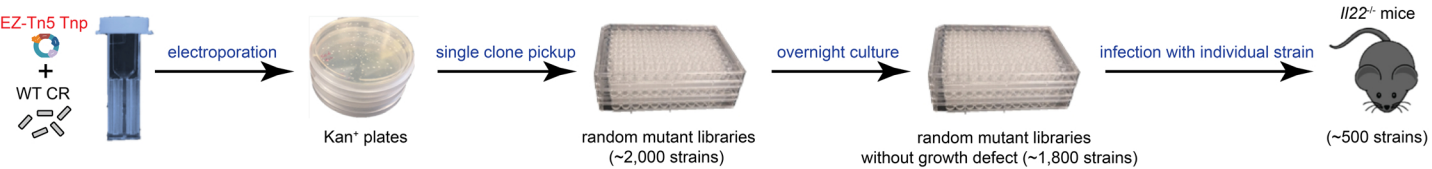

Supplement: S1 Fig — Wild-type CR transfected with EZ-Tn5-KAN-2 Transposome were recovered in SOC medium and plated onto kanamycin+ LB agar plates. All colonies (~2,000 strains) were picked and propagated in 96-well plates in LB medium containing 50 μg/ml of kanamycin. After removing the mutants with obvious growth defects, approximate 1,800 Tn5 mutant strains were divided into 18 sub-libraries (~100 strains each). The CR mutants from the first 5 sub-libraries (A-E) and wild-type CR were cultured overnight, and Il22-/- mice (3–5 animals for each individual strain) were infected by oral gavage with 2 × 10^9^ CFU of each individual strain (~500 strains). The clinical symptoms and survival of Il22-/- mice were monitored post infection for 28 days. (PDF) [file ppat.1007898.s004.pdf]

**A**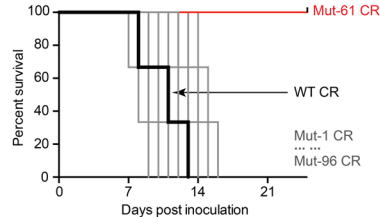**B**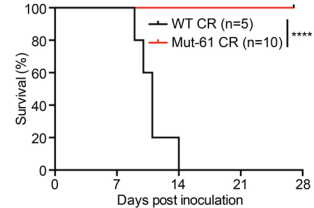**C**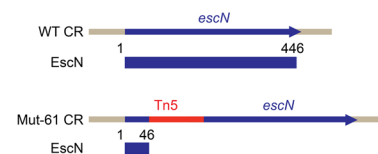**D**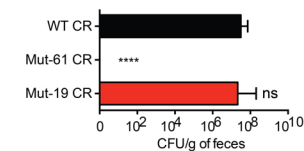

Supplement: S2 Fig — A. Kaplan-Meier analysis of the survival rates in Il22-/- mice inoculated with wild-type (WT) CR or each strain in the sub-library D consisting of 96 mutants (Mut-1 to Mut-96). B. Kaplan-Meier analysis of the survival rate in Il22-/- mice inoculated with WT or Mut-61 CR. C. Schematics of normal or Tn5-interrupted escN genes and EscN protein expression in WT and Mut-61 CR, respectively. D. The colony formation units (CFU) of live CR derived from fecal samples of Il22-/- mice, inoculated with 2 × 10^9^ CFU of indicated CR strains, at 7 dpi. ns, not significant, **** p < 0.001 by Long-rank test (B) and with one-way analysis of variance, followed by Bonferroni’s multiple comparison tests (D). (PDF) [file ppat.1007898.s005.pdf]

**A**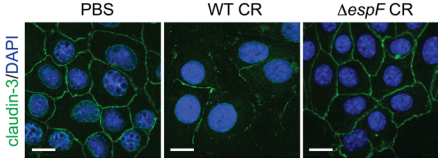**B**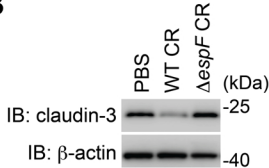

Supplement: S3 Fig — A. Representative immunofluorescence micrographs of CMT-93 cells infected in suspension with PBS, WT or ΔespF CR at 100 MOI for 3h, with nuclei counterstained by DAPI. Scale bars, 20 μm. B. CMT-93 cells were infected as in (A) and whole cell lysates were derived and immunoblotted (IB) for claudin-3, with β-actin as a loading control. (PDF) [file ppat.1007898.s006.pdf]

**A**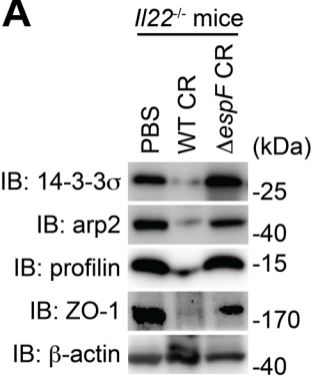**B**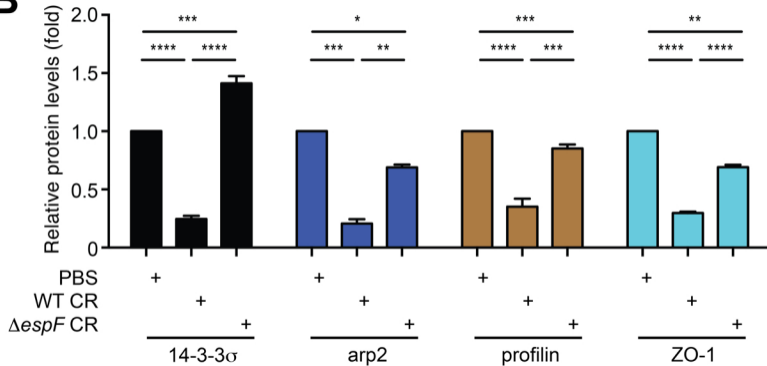

Supplement: S4 Fig — A. Il22-/- mice, inoculated with phosphate-buffered saline (PBS), wild-type (WT) CR, or ΔespF CR, and euthanized at 7 days post inoculation (dpi). Colon epithelial cell lysates were derived and immunoblotted (IB) for indicated proteins, with β-actin as a loading control. B. The indicated protein levels, normalized to β-actin and PBS controls, were quantified by ImageJ software from three independent experiments. * p < 0.05, ** p < 0.01, *** p < 0.001, and **** p < 0.0001 with one-way analysis of variance, followed by Bonferroni’s multiple comparison tests. (PDF) [file ppat.1007898.s007.pdf]

**A**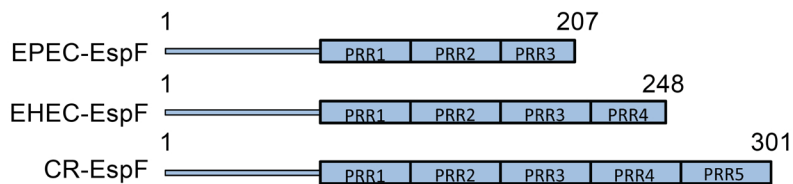**B**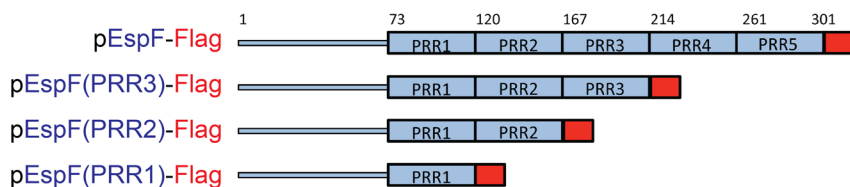**D**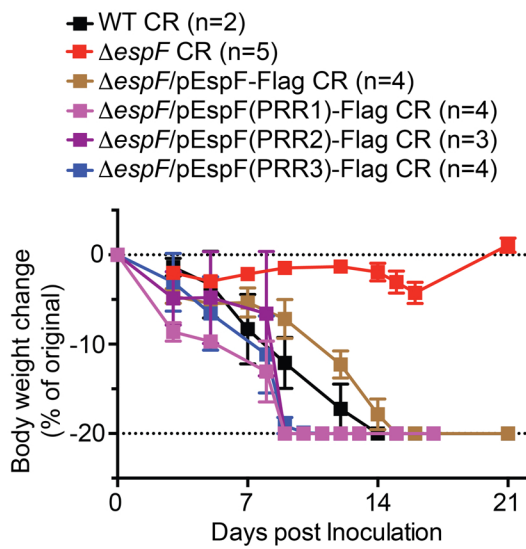**C**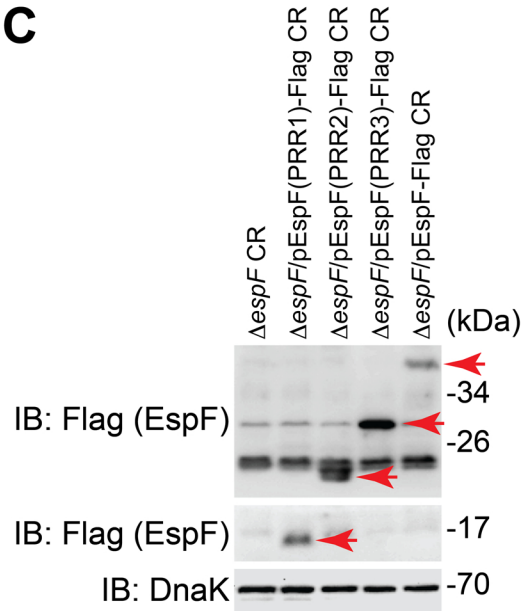**E**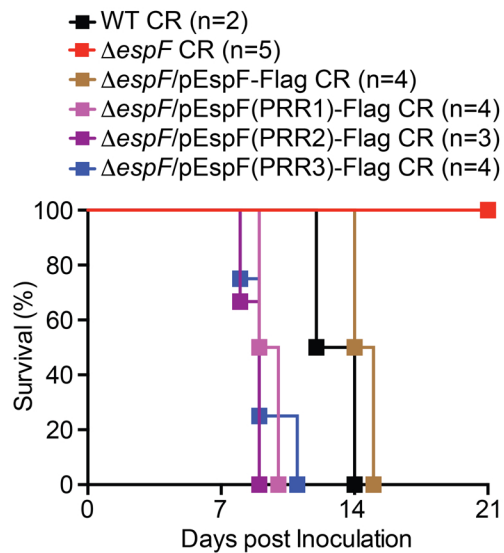

Supplement: S6 Fig — A. Schematic diagram of the EspF proteins from EPEC, EHEC, and CR. PRR, proline-rich repeat. B. Schematic diagram of full-length CR-EspF and indicated truncated CR-EspF containing different PRRs, fused with a C-terminal Flag tag. C. Whole cell lysates derived from the indicated CR strains were immunoblotted (IB) for Flag, with DnaK as a loading control. The full-length and truncated EspF-Flag proteins are marked with arrows. D. Weight loss of Il22-/- mice at indicated periods post inoculation with the indicated CR strains. E. Kaplan-Meier analysis of the survival rate in Il22-/- mice inoculated with the indicated CR strains. (PDF) [file ppat.1007898.s009.pdf]

**A**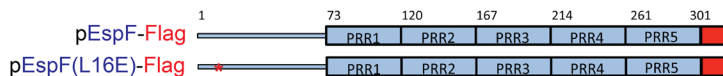**B**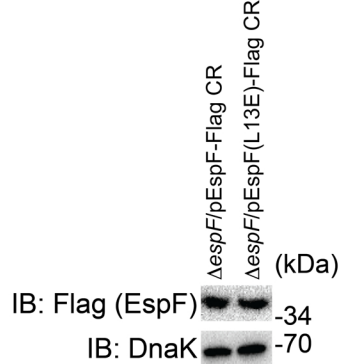**C**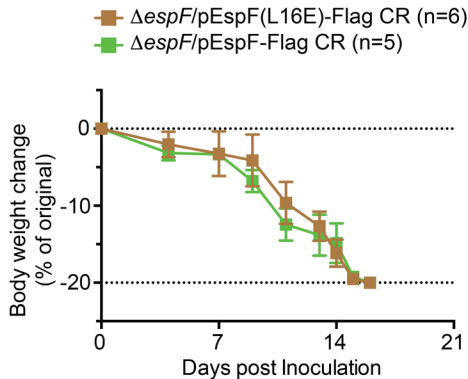**D**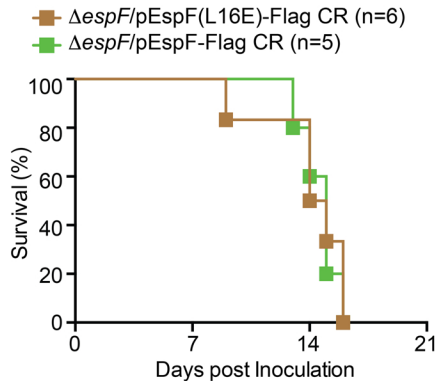

Supplement: S7 Fig — A. Schematic diagram of full-length and L16E mutant CR-EspF, fused with a C-terminal Flag tag. PRR, proline-rich repeat. B. Whole cell lysates derived from the indicated CR strains were immunoblotted (IB) for Flag, with DnaK as a loading control. C. Weight loss of Il22-/- mice at indicated periods post inoculation with the indicated CR strains. E. Kaplan-Meier analysis of the survival rate in Il22-/- mice inoculated with the indicated CR strains. (PDF) [file ppat.1007898.s010.pdf]
