# Supplementary material for: EspF is crucial for Citrobacter rodentium-induced tight junction disruption and lethality in immunocompromised animals
Source: PLoS Pathog. 2019 Jun 28;15(6):e1007898. doi: 10.1371/journal.ppat.1007898 (PMC6623547; doi:10.1371/journal.ppat.1007898)
Supplement: S5 Fig — A. Weight loss of Il22-/- mice at indicated periods post inoculation with 2 × 10^9^ CFU of mixed CR, with a ratio of wild-type (WT) CR to ΔespF CR at 1:1. B. Kaplan-Meier analysis of the survival rate in Il22-/- mice inoculated with mixed WT CR and ΔespF CR as in (A). C. The CR burden in the liver and the spleen derived from Il22-/- mice, infected as in (A) at 7 days post infection (dpi), were quantified. D. Live CR colonies were derived from the mixed inoculum at 0 dpi, or the liver and the spleen of infected Il22-/- mice at 7dpi. Individual CR colony was pickup and subjected to PCR-based identification as WT CR or ΔespF CR (as illustrated in Fig 2A). Shown are percentages of WT CR and ΔespF CR in the indicated numbers of live CR colonies examined. (PDF) [file ppat.1007898.s008.pdf]

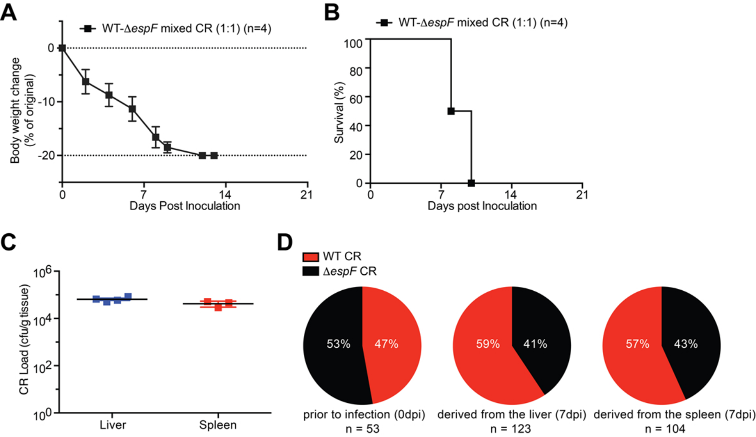
A
WT-ΔespF mixed CR (1:1) (n=4)
Body weight change (% of original)
0
-10
-20
0
7
14
21
Days Post Inoculation
B
WT-ΔespF mixed CR (1:1) (n=4)
Survival (%)
100
80
60
40
20
0
0
7
14
21
Days post Inoculation
C
CR Load (cfu/g tissue)
10^6
10^4
10^2
10^0
Liver
Spleen
D
WT CR
ΔespF CR
53%
47%
59%
41%
57%
43%
prior to infection (0dpi)
n = 53
derived from the liver (7dpi)
n = 123
derived from the spleen (7dpi)
n = 104
